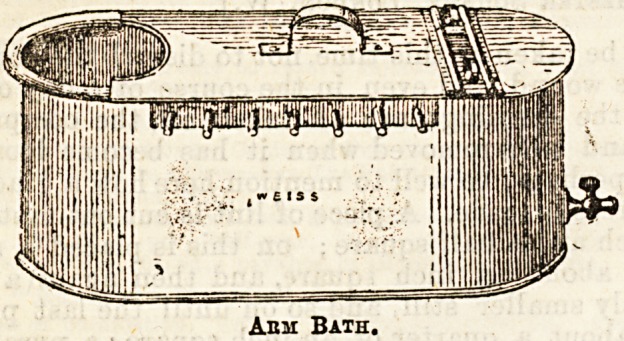# The Treatment of Injuries to the Hand

**Published:** 1892-12-31

**Authors:** 


### The Hospital Clinic.

[*The Editor will be glad to receive offers of co-operation and contributions from members of the profession. All letters should be addressed to* The Editor, The Lodge, Porchester Square, London, W.]

### THE LONDON HOSPITAL.

### The Treatment of Injuries to the Hand.

In speaking of the treatment of injuries to the hand, we shall deal with the subject under the following heads, viz., Wounds and their complications, Fractures, and Dislocations.

I. *Wounds.*—The number of simple wounds of the hand treated at a large hospital like the London is very great. By far the larger number of them are treated as out-patients, being dressed in the receiving room by a dresser, under the supervision of one of the two receiving room officers during the day, or one of the house surgeons at night. The treatment is simple; the wound is cleansed with some antiseptic lotion (corrosive sublimate or carbolic acid); if any arteries be pumping, they are taken up and tied with catgut, oosing is controlled by pressure, and then the edges of the wound are brought together with catgut or horsehair sutures, and a pad of boracic lint applied, over which is placed some absorbent wool and a bandage. If the wound be large, and the patient is likely to move it much, a simple straight splint is applied to the flexor aspect of the hand and forearm, and the whole placed in a sling. The subsequent treatment of these cases then goes into the hands of the surgical clinical assistant, who sits to see patients every afternoon in a room specially provided for him. If a wound, as above described, has opened a joint, the treatment is the same, excepting that perhaps a little more trouble is taken in cleansing, and instead of closing it tightly with sutures an opening is left for drainage. Should one of the tendons, either in the finger or back of the hand, be divided, the two ends are carefully sutured together; if they lie obviously in the wound this is quite easy, but usually the end attached to the muscle has retracted far into its sheath, so that some difficulty is encountered in finding it, and it may be necessary to enlarge the wound, and perhaps to give an anæsthetic. When found, the two ends are sutured together with catgut, the wound made thoroughly clean and then dressed, and the hand put on a splint. Should the injury be extensive, the patient is of course taken into the hospital. We have mentioned the ordinary treatment of arterial bleeding, but in some cases a wound of the *deep palmar arch* occurs, in which case the difficulty of applying a ligature to the bleeding vessel is very great, and would involve opening up the palm to such an extent that the hand would be much crippled in consequence. In these cases, the bleeding is controlled by judiciously applied pressure in the following manner: The bleeding is temporarily stopped by pressure on the brachial, while the wound is thoroughly cleansed, and a graduated compress is then made of boracic lint, and its apex accurately applied to the seat of bleeding. This is done by using a simple straight splint on the extensor aspect of the arm and hand and then bandaging over it. Before, however, putting on the splint, the fingers and arm are bandaged from below upwards to prevent subsequent swelling. The patient is then put in bed with the limb raised and the elbow flexed. Careful watch is now kept on the condition of the arm, as owing to the bandaging, which is somewhat tight, gangrene might occur if this were not looked out for by examination of the condition of the circulation in the tips of the fingers. Generally the bandages which surround the arm and fingers must be loosened at the end of eight or twelve hours; but the greatest care must be taken at this time, not to disturb the compress on the wound, and even in the course of a day or two, when the dressings are all taken off, the compress is left, and only removed when it has become loose. It may, perhaps, be well to mention here how a graduated compress is made. A piece of lint is cut out first about an inch and a half square; on this is placed a second piece about an inch square, and then again a third, slightly smaller still, and so on until the last piece is only about a quarter of an inch square; a pyramid is thus formed, the apex of which is applied to the bleeding point. In order to get a firmer base, this is sometimes made of a piece of cork or a coin wrapt in lint.

In many cases, however, the injuries which are brought to the hospital are more severe than simple wounds of the fingers, the hand being often crushed and mangled by being caught in moving machinery. In these cases one cardinal principle is always kept in view, namely, to remove as little of the hand as possible, so that what is most commonly done is to merely trim the stump, and bring the edges together as cleanly as possible. This is especially important in the thumb, every bit of which is most useful to oppose the fingers. Should an amputation of the fingers become necessary, this is usually done either through the second phalanx or at the metacarpo-phalangeal joint. It is advised at the London that the flaps should always be cut from without inwards (and not by transfixion), and that the incision be placed well on to the side of the finger, so as to make sure that the digital artery is left in the flap, which otherwise, being deprived of its blood supply, runs a grave risk of sloughing. The tendons, too, are stitched together over the end of the bone.

The most common subsequent *complication* of wounds in the hand is suppuration, and this is hardly to be wondered at when we think of the dirty condition of the part before the injury is received, in most cases, and the impossibility of thoroughly cleaning it. If suppuration occur, all stitches which are in any way tense, or likely to prevent free drainage, are removed, and then a warm dressing is applied in the form of boracic acid fomentations. These are easily made by soaking boracic lint or ordinary absorbent wool in a hot solution of boracic acid (ten grains to the ounce), or boro-glyceride (ʒi. to the pint). This is then applied to the wound hot, and over it is placed some form of waterproof tissue, and the whole is surrounded by a thick layer of cotton wool, to keep it warm as long as possible. This form of treatment, energetically carried out and early applied, often speedily put an ends to the suppurative process. In some cases, however, the suppuration spreads extensively, and may reach the palm from the fingers, more especially from the thumb and little fingers; or the wound may have originally opened up the palm. In these cases the freest drainage possible is what is especially aimed at, and with this end in view the wound is well opened up, and if by this means enough room for the escape of fluid is not made, a probe is passed, in the direction in which the pus is burrowing, and the end of it having been made to protrude, it is cut down upon, and so a counter opening is furnished, and then a drainage tube is passed through from one opening to the other. Care, of course, is taken to avoid any large vessels about the palm in making an opening of this kind. The subsequent treatment is to apply clean moisture and warmth, in the shape of a fomentation as above described. Another very common and most valuable method is to use a bath into which the forearm and hand can be placed, either continuously, or for several hours during the day. By this means the accumulating discharge is continuously washed away and diluted, and at the same time the warmth and moisture which are necessary are conveniently applied. The bath simply contains either plain water, kept

ARM BATH.

comfortably warm, or boracic acid lotion, or weak carbolic or iodine and water. During the intervals of rest from the bath fomentations are used. It is in these cases that cellulitis sometimes supervenes, spreading up the arm. When this occurs, the treatment adopted is to make free incisions into the cellular tissue, wherever there is any appearance of tension, and these allow the escape of the inflammatory products, and prevent sloughing; after these are made fomentations and the arm-bath are continued as before.

In all these cases when there has been much suppurative inflammation about the tendon sheaths, after the process has subsided and the wounds healed, the adhesions which are formed cause much stiffness of the fingers and hand, and in order to overcome this forcible passive movement is undertaken to break them down, and this is accompanied by massage and hot bathing. By these means much more movement is often obtained than might be expected.

II. The treatment of *fractures* about the hand will not detain us long. Fracture of a metacarpal bone is usually treated either by a straight splint which extends up the arm, and applied either on the dorsal or palmar aspect of the limb, whichever seems most convenient to the particular case; usually the latter is preferred. Or, instead of this form of splint, one is moulded to fit the part out of the patent poroplastic material, which can be made soft by heat, and then sets again hard and rigid so soon as it gets cold, or guttapercha is used in the same way. These are the materials, too, out of which splints are made for use in fractures of the phalanges; they are easily padded by means of a folded piece of lint.

Should the fracture be compound the wound is treated in the ordinary way, but before dressing it the greatest care is taken to make it aseptic if possible. These fractures usually unite well, but if suppuration takes place it is often rather prolonged before it quiets down.

III. *Dislocations* of the fingers are reduced, as a rule, with the greatest possible ease by merely a little well directed extension and manipulation, and the same is usually the case in displacement of the metacarpal bone of the thumb. Luxation of the proximal phalanx of the thumb, however, often causes a good deal of difficulty before it is returned into position. In the first place, the part to catch hold of is short, this, however, is easily overcome by using the thumb forceps. A firm grasp having been obtained, extension is first made in the direction of the axis of the displaced phalanx, which after a little is then steadily flexed, and at the same time, by manipulation of the head of the metacarpal bone and the long flexor tendon, which is sometimes in the way, the bone is replaced. The difficulties are sometimes so great, however, that the lateral ligaments of the joint have to be divided. If the dislocation be compound the bone is replaced, and the wound, after being made aseptic, is treated on ordinary principles, a splint being first applied to prevent movement or a return of the displacement.

## Figures and Tables

**Figure f1:**